# Distribution Characteristics of Suspended Macroalgae in the Southern Yellow Sea Before the Green Tide Outbreak

**DOI:** 10.3390/biology14101347

**Published:** 2025-10-02

**Authors:** Weimin Yao, Yaoyao Lei, Shulin Tan, Yutao Qin, Huanhong Ji, Yuqing Sun, Jianheng Zhang, Jinlin Liu

**Affiliations:** 1 Key Laboratory of Marine Ecological Monitoring and Restoration Technologies, East China Sea Ecological Center, Ministry of Natural Resources, Shanghai 201206, China; ywm@ecs.mnr.gov.cn (W.Y.); qinyutaox@163.com (Y.Q.); jihuanhong@163.com (H.J.); 2 College of Oceanography and Ecological Science, Shanghai Ocean University, Shanghai 201306, China; leiyaoyao2002@163.com (Y.L.); tanshulin666@gmail.com (S.T.); 3 State Key Laboratory of Marine Geology, Tongji University, Shanghai 200092, China; jlliu@tongji.edu.cn; 4 Project Management Office of China National Scientific Seafloor Observatory, Tongji University, Shanghai 200092, China

**Keywords:** green macroalgae, *Ulva*, harmful algal blooms, Yellow Sea, environmental factor

## Abstract

**Simple Summary:**

Green tides have become a widespread ecological concern around the world, with the Southern Yellow Sea (SYS) in China experiencing large-scale events dominated by *Ulva prolifera*. A total of 31 and 38 sampling stations were established in the SYS in 2023 and 2024, respectively. At these stations, environmental parameters, the abundance of suspended macroalgae, and species composition were analyzed. The study revealed that suspended macroalgae were predominantly distributed in the surface waters of nearshore areas, showing a positive correlation with nutrient levels. The main species identified were *U. prolifera* O.F.Müller, *Ulva linza* Linnaeus, *Ulva flexuosa* Wulfen, and *Blidingia* sp. Investigating the distribution characteristics of suspended macroalgae filaments during green tide outbreaks can substantially enhance our understanding of the underlying mechanisms driving these events.

**Abstract:**

For nearly two decades, the Yellow Sea has experienced recurrent green tides, which are now considered the largest of their kind globally; the mechanism behind these outbreaks remains highly complex and not fully understood. This study investigates the pre-outbreak distribution, abundance, and species composition of suspended macroalgae in the Southern Yellow Sea (SYS) during 2023–2024, along with environmental parameters. The results indicate that suspended macroalgae were predominantly distributed in the nearshore waters, particularly along the shallow beaches of northern Jiangsu. Furthermore, their abundance in the surface water layer significantly exceeded that in the bottom water. A total of 1353 and 493 algal filament samples were collected in 2023 and 2024, respectively. Dominant species included *Ulva prolifera*, *Ulva linza*, *Ulva flexuosa*, and *Blidingia* sp. Nutrient levels positively correlated with filament abundance. As a primary means of rapid proliferation for *U. prolifera*, suspended macroalgae contribute significantly to the initial expansion of green tides. Furthermore, their abundance holds promise as a biological indicator for forecasting the scale and extent of impending blooms, thereby providing a critical foundation for elucidating the underlying outbreak mechanisms.

## 1. Introduction

The increasing frequency and expanding scale of green tide outbreaks are primarily fueled by the synergistic effects of escalating seawater eutrophication and global climate change [1,2,3,4,5,6]. Green tide events, systematically documented across coastal regions of multiple countries [7,8,9,10,11,12], collectively underscore their severity as a widespread environmental challenge. The Southern Yellow Sea (SYS) of China has experienced large-scale green tides for 19 consecutive years (2007–2025), making it one of the longest-lasting regional algal bloom events worldwide [13,14,15].

*Neopyropia yezoensis* (Ueda) L.-E. Yang & J. Brodie aquaculture rafts in the Subei Shoal are identified as one of the major sources providing the initial biomass for floating green-tide macroalgae in the SYS [16,17,18]. As the dominant species of green tides in the SYS [19], *Ulva prolifera* exhibits distinctive morphological and reproductive characteristics [20,21]. Owing to its morphological adaptations, including a hollow, monolayer tubular structure, extensive branching, and a pronounced ability to form gas vesicles, *U. prolifera* achieves positive buoyancy [22]. This allows it to float and form extensive mats on the sea surface through rapid reproduction and aggregation. *U. prolifera* exhibits a diversity of reproduction modes, namely sexual, asexual, and vegetative propagation [21,23]. The microscopic propagules of *Ulva* primarily include gametes, spores, zygotes, and vegetative fragments [24,25]. The gametes, spores, and zygotes of *U. prolifera* require suitable substrates for germination and growth, a requirement met by the aquaculture rafts used for *Neopyropia* cultivation in Jiangsu Province [26]. Under the suitable temperature, gametes of *U. prolifera* that survive from the previous year rapidly germinate, grow, and mature into gametophytes [27], thereby supplying the initial biomass for green tides in the SYS. Furthermore, during the mass proliferation of *U. prolifera*, the freely floating algal mats can provide additional substrate for the attachment of microscopic propagules of *U. prolifera* in the water column [28]. Fragmentation of tissue can rapidly induce the transformation of vegetative cells in *U. prolifera* into germ cells, thereby facilitating rapid biomass accumulation in the dominant green-tide species [29].

*Ulva prolifera* mainly relies on vegetative reproduction in the sea [30], fragments of *U. prolifera* thalli can develop into new individuals under suitable conditions. Zhang et al. [31] observed that small fragment of *U. prolifera* can develop new branches and rapidly grow up over a short period. Vegetative fragments of *U. prolifera* that settle from previous green tide events may form a potential “seed bank”, contributing to the initiation of subsequent outbreaks in the following year [23]. *Ulva* undergoes fragmentation, generating thallus fragments and broken branches as a result of external forces or natural growth processes. Suspended macroalgae refer to macroscopically visible *U. prolifera* seedlings and thallus fragments, typically ranging from approximately 0.5 cm to 10 cm in length, these filaments continue to grow, thereby supplying additional biomass to green tide events.

To investigate the role and contribution of suspended macroalgae in the initiation of green tides in the SYS, this study examines the spatiotemporal distribution and influencing factors of both the water column and suspended macroalgae in the outbreak areas of Jiangsu Province, aim to provide a theoretical foundation for the prevention and mitigation of green tides in the region.

## 2. Materials and Methods

### 2.1. Station Setup

**Figure 1:**
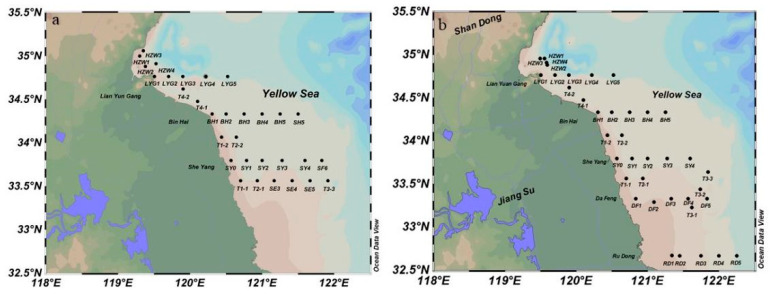
Distribution of survey stations in the Southern Yellow Sea. (**a**) Stations established in March 2023, (**b**) Stations established in March 2024.

Survey cruises were conducted in the SYS (32.0° N–36.0° N, 119.0° E–123.0° E) during March 2023 and March 2024. In March 2023, a total of 31 sampling stations were established based on the hydrographic and environmental features of the study area (Figure 1a). The sampling effort was expanded to 38 stations in March 2024 (Figure 1b). Each identified by station number and cruise number (Table 1). Both planned and measured geographical coordinates (latitude and longitude) were documented to ensure comprehensive spatial coverage of representative subregions.

### 2.2. Environmental Parameter Measurement

Water samples were collected at various depths, including the surface layer (0.5 m) and the bottom layer (within 2 m above the seabed), from each sampling station. At each site, three replicate samples were taken using polyethylene bottles that had been pre-cleaned with deionized water and rinsed with in situ seawater prior to sampling. The pH, temperature, and salinity were measured in situ using a portable water quality analyzer (DZB-712F, LeiCi Co., Ltd., Shanghai, China).

The analyzed parameters included ammonium nitrogen (NH_4_^+^-N), nitrate nitrogen (NO_3_^−^-N), nitrite nitrogen (NO_2_^−^-N), dissolved inorganic nitrogen (DIN), phosphate (PO_4_^3−^-P), and silicate (SiO_3_^2−^-Si). Concentrations of NH_4_^+^-N, NO_3_^−^-N, and NO_2_^−^-N were determined spectrophotometrically. DIN was calculated as the sum of NH_4_^+^-N, NO_3_^−^-N, and NO_2_^−^-N. Phosphate was measured using the molybdenum blue method, and silicate was determined using the silicate–molybdenum blue colorimetric method [32,33,34].

### 2.3. Suspended Macroalgae Sampling

Samples were collected following standardized protocols for phytoplankton sampling [35]. Suspended macroalgae were sampled using two methods: vertical towing and horizontal towing. Vertical tows were conducted by hauling a Shallow Water Type I plankton net (mouth diameter: 50 cm, mesh size: 505 µm) from the seabed to the surface. Horizontal tows included both surface and bottom types: surface tows were carried out at depths exceeding 1 m, while bottom tows were performed within 2 m above the seabed. Each tow lasted 15 min at a vessel speed of 2–3 knots. All samples were immediately preserved at −20 °C for subsequent analysis.

### 2.4. Suspended Macroalgae Counting and Processing

The collected suspended macroalgae were filtered through a 100-mesh screen to remove extraneous debris. The retained suspended macroalgae were transferred to an enamel pan and resuspended in a suitable amount of seawater. The abundance of suspended macroalgae was quantified for each sampling station. Based on morphological features, the filaments were preliminarily classified into broad taxonomic groups. Finally, the algal material was rinsed with sterilized seawater in preparation for molecular biological identification.

Genomic DNA was extracted from the algal samples using the Dzup (Plant) Genomic DNA Isolation Reagent (Sangon Biotech, Shanghai, China). Polymerase chain reaction (PCR) amplification was conducted targeting the internal transcribed spacer (ITS) region and the 5S ribosomal DNA (5S rDNA) intergenic spacer, with specific primers designed for the purpose (Table 2). The qualified PCR products were subsequently sent for sequencing at Sangon Biotech Co., Ltd. (Shanghai, China).

The PCR amplification protocol for the ITS region was carried out under the following conditions: initial denaturation at 94 °C for 5 min; followed by 30 cycles of denaturation at 94 °C for 40 s, annealing at 55 °C for 40 s, and extension at 65 °C for 70 s; with a final extension at 65 °C for 10 min.

The PCR amplification protocol for the 5S rDNA intergenic spacer was performed as follows: initial denaturation at 94 °C for 3 min; followed by 35 cycles of denaturation at 94 °C for 40 s, annealing at 52 °C for 40 s, and extension at 72 °C for 70 s; with a final extension at 72 °C for 5 min.

The target gene sequences were obtained by Sanger sequencing. After manual correction of sequencing peaks, the sequences were preliminarily identified by similarity comparison using the BLAST+ 2.17.0 tool in the National Center for Biotechnology Information database (NCBI; https://www.ncbi.nlm.nih.gov/). Reference sequences of related species were also downloaded from NCBI. Multiple sequence alignment was carried out with Clustal X 2.0 [39]. Based on the aligned sequences, genetic distances were computed using the Kimura two-parameter model, and a phylogenetic tree was reconstructed with the Maximum Likelihood method in MEGA 7.0. The reliability of the phylogenetic tree was evaluated using bootstrap analysis based on 1000 replicates [40,41].

## 3. Results

### 3.1. Changes in Environmental Factors

#### 3.1.1. Changes in Physical and Chemical Parameters

**Figure 2:**
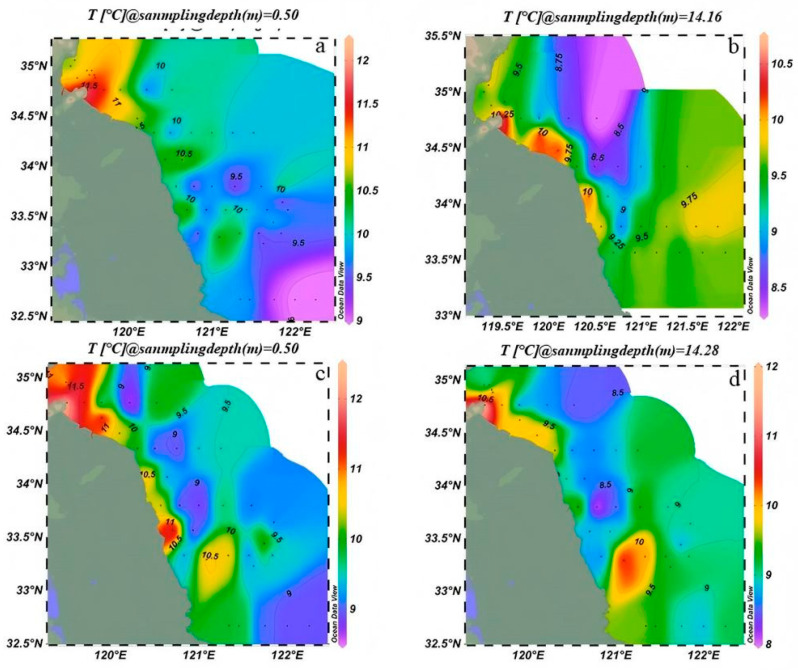
Changes in sea surface temperature in the Southern Yellow Sea in 2023 and 2024. (**a**) surface water temperature in March 2023, (**b**) bottom water temperature in March 2023, (**c**) surface water temperature in March 2024, (**d**) bottom water temperature in March 2024.

From March 2023 to March 2024, sea surface temperature (SST) in the SYS exhibited a general decreased trend from nearshore to offshore regions, with notable interannual and vertical variations. In March 2023, surface waters (Figure 2a) were relatively cool, with nearshore shallow areas reached up to 12 °C, while offshore waters averaged ~9 °C, indicating a clear onshore–offshore gradient. Bottom-layer temperatures were lower than those at the surface, averaging ~10 °C in coastal areas and ~8.5 °C offshore, with a comparatively weaker gradient (Figure 2b). In March 2024, temperatures increased throughout the region. Surface waters (Figure 2c) exceeded 12 °C nearshore and reached ~10.1 °C offshore. Bottom-layer temperatures (Figure 2d) followed a similar pattern, rising to ~11 °C nearshore and ~9.5 °C offshore. As a result, the vertical temperature difference between surface and bottom layers was reduced relative to 2023.

From March 2023 to March 2024, notable differences in salinity, pH, dissolved oxygen (DO), and chemical oxygen demand (COD-Mn) were observed between surface and bottom layers in the SYS, reflecting the spatiotemporal characteristics of the spring hydrographic environment (Table 3).

In March 2024, mean salinity values were 29.54 ± 1.99 at the surface and 29.89 ± 1.40 at the bottom, both lower than the corresponding values in March 2023 (surface: 30.42 ± 1.12; bottom: 30.41 ± 1.09). Surface and bottom pH in March 2024 were 8.07 ± 0.06 and 8.08 ± 0.06, slightly lower than those in March 2023 (surface: 8.14 ± 0.05; bottom: 8.15 ± 0.05), while remained weakly alkaline. Dissolved oxygen concentrations increased in 2024, averaged 292.48 ± 5.32 μmol/L at the surface and 296.46 ± 4.84 μmol/L at the bottom, compared with 280.84 ± 8.47 μmol/L and 295.18 ± 6.91 μmol/L in 2023. COD-Mn also increased, with surface and bottom values of 29.38 ± 11.60 μmol/L and 34.93 ± 15.76 μmol/L in 2024, higher than 16.14 ± 7.91 μmol/L and 23.36 ± 16.74 μmol/L in 2023, respectively.

In 2023, salinity in both surface and bottom layers remained at relatively high levels, with no significant vertical differences. By contrast, in 2024, surface salinity decreased while bottom salinity was relatively higher, showing a stratification pattern, and the overall salinity was lower than in 2023. The pH values in 2023 exhibited little difference between surface and bottom layers, whereas in 2024 the surface pH increased markedly, with a relatively limited rise in the bottom layer, resulting in generally higher pH levels compared to 2023. Dissolved oxygen (DO) in 2023 was slightly higher in the surface layer than in the bottom layer, with only minor differences; however, in 2024, surface DO increased substantially, and bottom DO also rose, leading to an overall higher DO concentration than in 2023. COD-Mn remained at relatively high levels in both surface and bottom waters in 2023, but declined in both layers in 2024. In summary, the water column in 2023 was characterized by high salinity and high COD-Mn, accompanied by relatively lower pH and DO, whereas in 2024 it exhibited lower salinity and COD-Mn, together with elevated pH and DO.

#### 3.1.2. Interannual Variation of Nutrients in the Southern Yellow Sea

**Figure 3:**
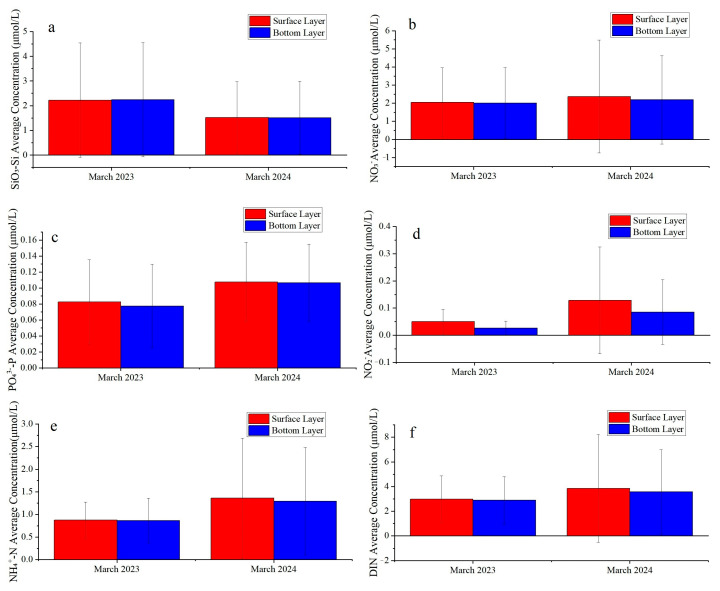
Temporal variation of nutrients in the Southern Yellow Sea. (**a**) SiO_3_−Si; (**b**) NO_3_^−^; (**c**) PO_4_^3−^−P; (**d**) NO_2_^−^; (**e**) NH_4_^+^−N; (**f**) DIN.

In March 2023, silicate (SiO_3_^2−^-Si) concentrations were relatively high in both surface and bottom layers, averaged close to 2 μmol/L. In March 2024, concentrations decreased to approximately 1.5 μmol/L (Figure 3a). Nitrate nitrogen (NO_3_^−^-N) averaged ~2 μmol/L in both layers in 2023 and showed a slight increase in 2024 (Figure 3b). Phosphate (PO_4_^3−^-P) concentrations increased markedly in March 2024, with similar levels in surface and bottom waters, representing a notable rise compared with 2023 (Figure 3c).

Nitrite nitrogen (NO_2_^−^-N) concentrations were generally low (<0.5 μmol/L) in 2023 but increased noticeably in March 2024, especially in the surface layer, reaching nearly 2 μmol/L (Figure 3d). Bottom-layer ammonium nitrogen (NH_4_^+^-N) also increased in 2024 relative to 2023 (Figure 3e). Consequently, dissolved inorganic nitrogen (DIN, the sum of NO_3_^−^-N, NO_2_^−^-N, and NH_4_^+^-N) concentrations in both layers were higher in 2024, averaging around 2–4 μmol/L, (Figure 3f).

#### 3.1.3. Spatial Distribution of Phosphate and Dissolved Inorganic Nitrogen in the Southern Yellow Sea

**Figure 4:**
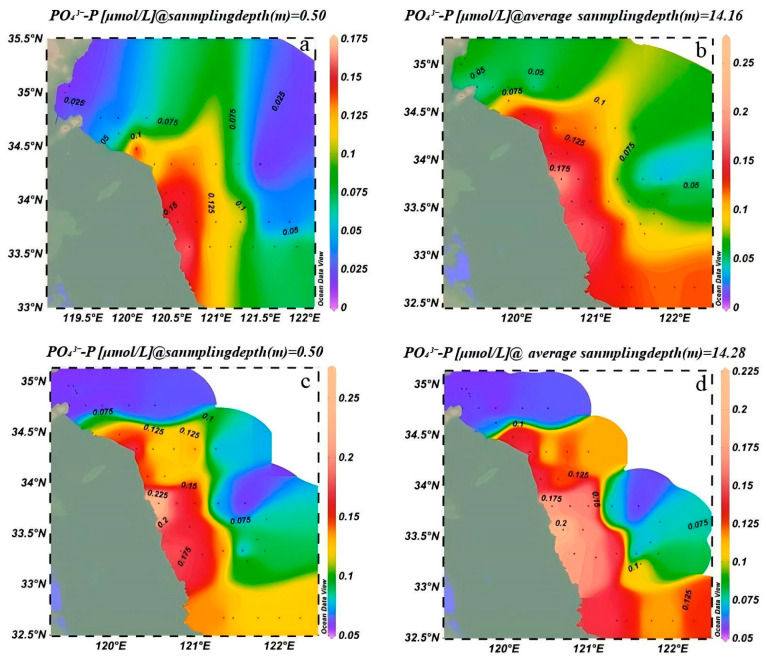
Spatial distribution of phosphorus nutrients in the Southern Yellow Sea. (**a**) Surface PO_4_^3−^-P in March 2023, (**b**) Bottom PO_4_^3−^-P in March 2023, (**c**) Surface PO_4_^3−^-P in March 2024, (**d**) Bottom PO_4_^3−^-P in March 2024. (The surface sampling depth is 0.5 m, and the bottom sampling depth is 2 m from the bottom layer).

In March 2023, the range of surface concentrations of PO_4_^3−^-P was 0.025–0.175 μmol/L. Areas with high concentrations (≥0.15 μmol/L) were found in nearshore waters between 34.5° N and 35° N and 119.5° E and 121° E. These concentrations decreased to below 0.05 μmol/L in offshore waters (Figure 4a). The bottom-layer concentration of PO_4_^3−^-P ranged from 0.025 to 0.175 μmol/L. High-value zones (≥0.15 μmol/L) were confined to the nearshore area between 34.5° N and 35° N. Offshore areas (south of 33° N) had concentrations as low as 0.025 μmol/L (Figure 4b).

In March 2024, surface concentrations of PO_4_^3−^-P increased significantly by 0.05–0.25 μmol/L, accompanied by an expansion of the high-value zone. New high-concentration centers exceeded 0.25 μmol/L appeared north of 35.5° N and east of 122° E, showed a pronounced onshore–offshore gradient with higher values near the coast and lower values offshore (Figure 4c). Bottom-layer PO_4_^3−^-P concentrations also increased, ranged from 0.05 to 0.25 μmol/L. New high-concentration centers (~0.25 μmol/L) emerged north of 35.5° N, and concentrations generally rose to 0.10–0.15 μmol/L in the 33.5–34° N region, indicated substantial bottom-layer phosphate replenishment (Figure 4d).

**Figure 5:**
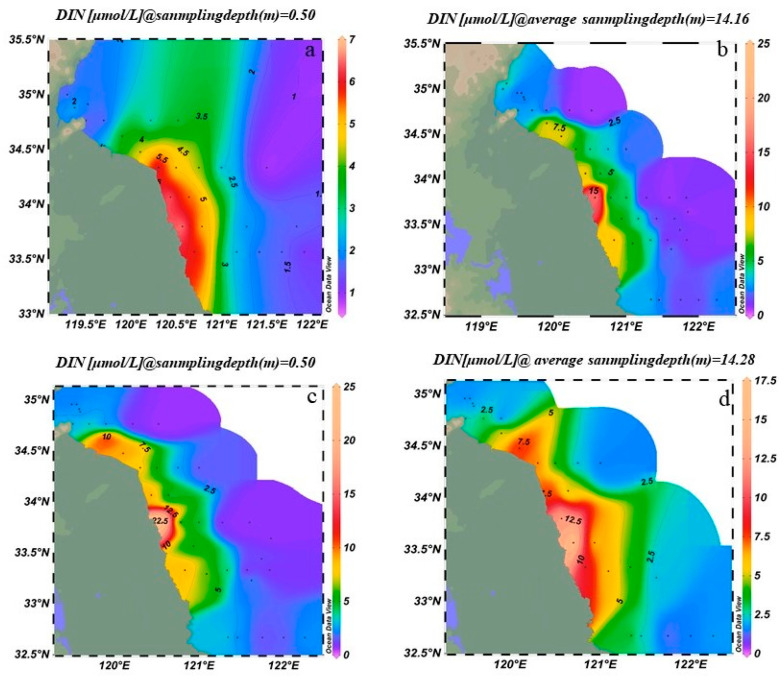
Spatial distribution of nitrogen nutrients in the Southern Yellow Sea. (**a**) surface DIN in March 2023, (**b**) bottom DIN in March 2023, (**c**) surface DIN in March 2024, (**d**) bottom DIN in March 2024. (The surface sampling depth is 0.5 m, and the bottom sampling depth is 2 m from the bottom layer).

In March 2023, surface concentrations of DIN ranged from 1 to 7 μmol/L. High-concentration zones (≥5 μmol/L) were located between 34° N–35° N and 120° E–121° E, whereas values dropped sharply to below 1 μmol/L in the open sea east of 122° E (Figure 5a). Bottom-layer DIN concentrations ranged from 2 to 8 μmol/L, with high-concentration zones (≥6 μmol/L) between 34.5° N and 35° N, and lower concentrations (~2 μmol/L) in the open sea south of 33° N (Figure 5b).

In March 2024, surface DIN concentrations ranged from 2 to 25 μmol/L. The main high-concentration zone (≥20 μmol/L) extended northeastward to 35.5° N, and a secondary high (~10 μmol/L) appeared offshore at 123° E (Figure 5c). The previously extensive 10–15 μmol/L zone contracted, while another secondary high (~8 μmol/L) emerged between 34° N and 34.5° N. Bottom-layer DIN exhibited a “coastal reduction and offshore local accumulation” pattern, with concentrations ranging from 2 to 20 μmol/L, and local offshore highs around 15–20 μmol/L (Figure 5d).

### 3.2. Spatiotemporal Variation in Suspended Macroalgae Abundance in the Southern Yellow Sea

#### 3.2.1. Variation in Suspended Macroalgae Abundance

**Figure 6:**
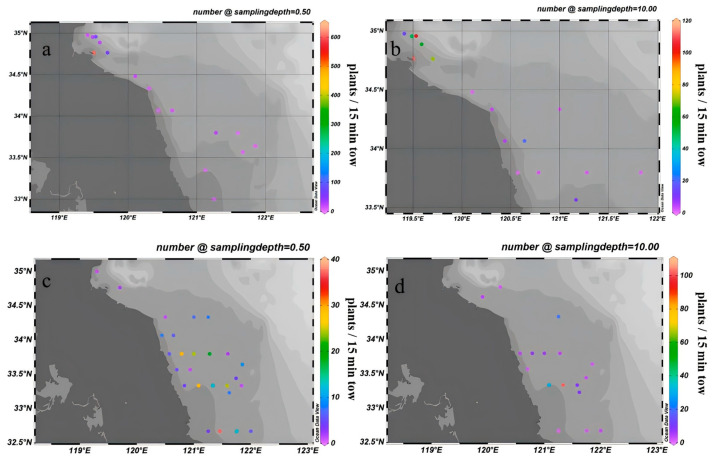
Abundance of suspended macroalgae in surface (**a**) and bottom (**b**) waters of the study area in March 2023, and surface (**c**) and bottom (**d**) waters in March 2024.

According to horizontal tow net data from 2023, the total abundance of suspended macroalgae in surface waters was 900 fragments, with the majority concentrated at station LYG1 (606 fragments) (Figure 6a). In bottom waters, the total was 453 fragments, mainly distributed at stations LYG1 (112 fragments), LYG2 (70 fragments), and HZW2 (78 fragments) (Figure 6b). Data from 2024 revealed significant interannual variation: the surface total was 276 fragments, with high abundances recorded at RD2 (37 fragments), DF2 (29 fragments), and SY1 (29 fragments) (Figure 6c). The bottom total was 217 fragments, concentrated at DF3 (102 fragments), DF2 (30 fragments), and BH5 (21 fragments) (Figure 6d). Compared with 2023, the total abundance of suspended macroalgae decreased from 1353 to 493 fragments, a reduction of 63.6%, comprising a 69.3% decrease in surface waters and a 52.1% decrease in bottom waters. Additionally, vertical tow nets collected 8 suspended macroalgae fragments, sparsely distributed across 7 stations including SY0 and DF2.

#### 3.2.2. Variation in Suspended Macroalgae Abundance in the Radial Sand Ridges During 2024

**Figure 7:**
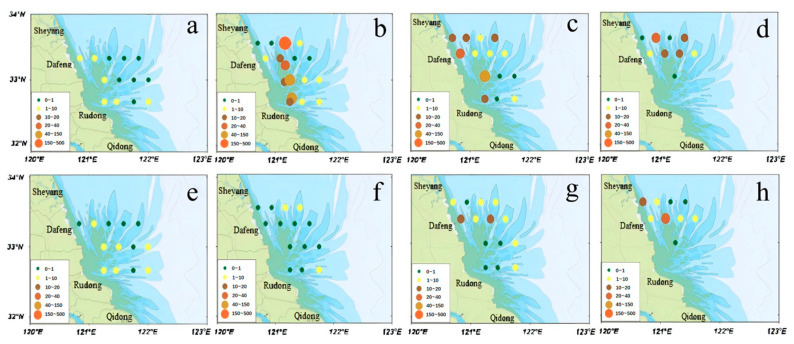
Abundance of suspended macroalgae in the waters of the Radial Sand Ridges from April to May 2024. (**a**–**d**) show the abundance of suspended macroalgae detected in surface waters during 14–19 April, 21–29 April, 12–22 May, and 24–31 May 2024, respectively; (**e**–**h**) show the corresponding abundance in bottom waters during 14–19 April, 21–29 April, 12–22 May, and 24–31 May 2024. Note: Abundance units are plants per 10 min tow.

During April to May 2024, the distribution of suspended macroalgae showed clear phased variations (Figure 7). From 14–19 April, suspended macroalgae abundance was sparse, ranging from 0 to 5 individuals per 10 min, and was relatively uniform in both surface and bottom waters across the study area (Figure 7a,e). Between 21 and 29 April, a significant proliferation occurred in surface waters, with abundances ranged from 0 to 500 individuals/10 min. Notably, high values were observed at stations SC1 and SE3 (Figure 7b,f), while the bottom waters remained at very low densities. In May, surface densities somewhat decreased, whereas the bottom layers showed a gradual accumulation of suspended macrosuspended macroalgae (Figure 7c,d,g,h). Overall, suspended macroalgae abundance in surface waters followed an initial increase followed by a decrease, with higher levels in late April and early to mid-May. In contrast, abundance in bottom waters increased slowly over time, showing partial correlation with the distribution of large-scale floating macroalgae mats.

### 3.3. Changes in the Spatiotemporal Distribution and Species Composition of Suspended Macroalgae in the Southern Yellow Sea

**Figure 8:**
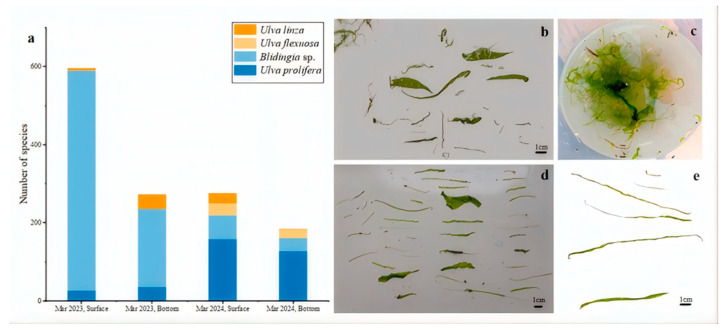
Species and morphology of suspended macroalgae. (**a**) Number of species; (**b**–**e**) Morphology of suspended macroalgae.

In March 2023, *Blidingia* sp. was the absolute dominant species in surface waters, accounting for 94.3% of the total community, while *U. prolifera* and *U. linza* constituted less than 5.6%, and *U. flexuosa* was completely absent (Figure 8a). In bottom waters, the abundance of *Blidingia* sp. decreased, whereas the proportions of *U. prolifera* and *U. linza* increased to 38.6%. By March 2024, the abundance of *U. prolifera* in surface waters had surged by 510.5%, replaced *Blidingia* sp. as the new dominant species. Meanwhile, *U. flexuosa* and *U. linza* appeared in significant quantities for the first time. This trend continued in bottom waters, with *U. prolifera* remained dominant, *Blidingia* sp. decreased sharply by 83.4%, *U. flexuosa* became the third most abundant species, and *U. linza* nearly disappeared (Figure 8b–e). Regarded vertical distribution, algal abundance was higher in surface waters than in bottom waters in both years. However, the surface-to-bottom difference narrowed in 2024, with surface abundance only 1.5 times that of the bottom layer, compared to 2.2 times in 2023.

The results revealed a strong correlation between the spatial distribution of suspended macroalgae and nutrient levels, particularly the concentrations of DIN and PO_4_^3−^-P. As shown in Figure 4 and Figure 5, high-concentration areas of DIN and PO_4_^3−^-P persisted in the nearshore waters of Jiangsu, especially in the northern region (34° N–35.5° N), during the springs of 2023 and 2024. These nutrient-rich zones spatially coincided with areas of high filament abundance (e.g., station LYG1 in 2023 and stations DF2, DF3 in 2024; Figure 6). This confirms that nearshore eutrophication from terrestrial inputs and aquaculture activities was a primary driver of the initial accumulation and growth of suspended macroalgae in shallow nearshore areas. Increased water temperature was another key factor influencing interannual variation in species composition. Compared to March 2023, water temperatures in the Jiangsu coastal area were generally 1–2 °C higher in March 2024 (Figure 2). This thermal change was particularly favorable for the growth of *U. prolifera*. This aligns with the dramatic increase (510.5%) in the proportion of *U. prolifera* observed in the species composition in 2024 (Figure 8). Regarding vertical distribution, the significantly higher abundance of suspended macroalgae in the surface layer compared to the bottom layer (Figure 6) was primarily attributed to their inherent buoyancy and the availability of sufficient light in the surface waters. However, the narrowing difference in abundance between surface and bottom layers in 2024 (decreasing from 2.2 times in 2023 to 1.5 times) might be related to the enhanced vertical mixing observed in 2024 (manifested as weakened stratification and reduced vertical temperature difference, Figure 2), which promoted vertical exchange of filaments within the water column.

## 4. Discussion

Temperature is a critical environmental factor influencing the growth and reproduction of macroalgae [42,43,44]. During spring, the sea temperature in the SYS exhibits a spatial pattern characterized by a general decrease from nearshore areas toward the open sea, with notable variations across different years and water layers. In March 2024, the coastal waters of Jiangsu exhibited slightly higher temperatures compared to the same period in 2023, accompanied by a noticeable reduction in the temperature difference between surface and bottom layers. This pattern indicates that spring warming enhances solar heating of the seawater, while intensified air–sea heat exchange and vertical mixing contribute to a more uniform temperature distribution throughout the water column. The microscopic propagules of *U. prolifera* begin to germinate at 5 °C. As a eurythermal species, it is capable of growing within a temperature range of 5–30 °C, with large-scale green tides typically forming around 17 °C [45,46,47]. In March, a correlation was observed between filament abundance and temperature, suggesting that rising temperatures may promote the growth of suspended macroalgae.

Concentrations of nitrogen and phosphorus nutrients exhibited a distinct cross-shelf gradient, characterized by higher levels in nearshore waters, particularly in the southwestern region off Jiangsu, and gradually decreasing concentrations toward the open sea and northern areas. The Subei Shoal is a well-documented area for nutrient enrichment, with consistently high concentrations of nitrogen and phosphorus observed in its nearshore waters [47,48,49]. These abundant nutrient supplies provide the essential material basis for the outbreak of green tides in the SYS [50,51,52]. Compared to March 2023, SiO_3_^2−^-Si decreased significantly in the spring of 2024, suggesting relatively lower primary productivity of diatoms. NO_3_^−^-N levels showed no significant difference between 2023 and 2024, indicating relatively stable sources of nitrate (such as terrestrial input and remineralization in the SYS during spring); however, localized enrichment in bottom waters still requires attention. In March 2024, the concentration of PO_4_^3−^-P increased significantly compared to March 2023, suggesting a stronger influence of exogenous phosphorus inputs on the SYS during the spring of 2024.

Microscopic propagules serve as “seeds” for macroalgae and play a significant role in the formation of green tides [53]. High abundances of microscopic propagules of *Ulva* sp. have been documented in both the water column and sediments of the SYS prior to and during green tide outbreaks, suggesting their potential role in the initiation and maintenance of these events [16]. Moreover, microscopic propagules of *Ulva* exhibit a strong capacity for overwintering and persisting through summer conditions [53,54,55]. Nutritive fragments and somatic cells of *Ulva* have been detected in sediments of the SYS. Under laboratory conditions, these structures demonstrate high rates of germination and growth, suggesting that overwintering fragments and cells may serve as potential seed sources for green tide initiation [56,57]. Prior to the outbreak of green tides, the density of microscopic propagules exhibited a gradual decline from the waters adjacent to nearshore raft aquaculture areas toward the eastern offshore and northern regions of the SYS [16,42]. This spatial pattern was consistent with the variations in suspended macroalgae abundance observed in both 2023 and 2024. The maximum coverage of the green tide was 998 km^2^ in 2023 and 591 km^2^ in 2024, representing a decrease of approximately 48% compared to the previous year according to monitoring data. In March 2024, the total abundance of suspended macroalgae decreased substantially compared to the same period in 2023, with reductions of 69.3% in the surface layer and 52.1% in the bottom layer. Overall, during the pre-outbreak period of green tides in the SYS, the abundance of suspended macroalgae shows a certain correlation with the magnitude of the green tide event in the same year [58].

The attached macroalgae on *Neopyropia* aquaculture rafts in the Subei Shoal are identified as a significant source of microscopic propagules in the water column [49]. Long-term field investigations have revealed that the dominant macroalgae species attached to *Neopyropia* aquaculture rafts in the Subei Shoal include *U. prolifera*, *U. linza*, *U. flexuosa*, and *Blidingia* sp. [59]. Meanwhile, the microscopic propagules in the Jiangsu coastal waters are primarily composed of *U. prolifera*, *U. linza*, *U. flexuosa*, *Ulva torta*, *Ulva simplex*, *Ulva californica*, and *Ulva meridionalis* [60]. The unique geophysical environment of the Subei Shoal (strong tidal waves, friction between suspended sediments and thalli, and gravity shearing when long thalli are exposed to air) facilitates the fragmentation of green macroalgae and the release of somatic cells [56]. Zhang et al. [61] reported that the natural detachment rate of attached green algae on *Neopyropia* aquaculture rafts ranged between 2.41% and 3.80%. During the pre-outbreak periods of the green tides in 2023 and 2024, the suspended macroalgae collected were largely consistent in species composition with the macroalgae attached to *Neopyropia* aquaculture rafts. Furthermore, the same species were also identified among the propagules present in both the water column and sediments. Therefore, the potential relationship between suspended macroalgae and *Neopyropia* rafts, as well as their contribution to green tide outbreaks in the Yellow Sea, require further investigation.

## 5. Conclusions

This study analyzed the abundance and species composition of suspended macroalgae, along with environmental factors such as nutrient concentrations and water temperature, prior to the green tide outbreaks in the Yellow Sea in 2023 and 2024 [62]. It further investigated the spatiotemporal characteristics and influencing factors of both the water column and suspended macroalgae in the outbreak areas off Jiangsu Province. The results indicate that, given sufficient nutrient availability in the Jiangsu coastal area, the rise in sea temperature observed in March 2024 may have contributed to the increase in the proportion of *U. prolifera*. Additionally, a correlation was observed between the distribution of suspended macroalgae and large-scale floating algal aggregates, which elucidates a critical “environment-physiology” response mechanism with direct applied value for predicting outbreaks, assessing aquaculture stress, and guiding nutrient-control strategies.

## Figures and Tables

**Table 1:** Station longitude and latitude information table.

Year	Station	Latitude (°N)	Longitude (°E)
2023	T3-3	121°54′49.80″	33°33′53.34″
	SE5	121°39′57.00″	33°33′53.34″
	SE4	121°25′04.80″	33°33′53.34″
	SE3	121°10′02.40″	33°33′53.34″
	T2-1	120°56′02.10″	33°33′53.34″
	T1-1	120°42′03.48″	33°33′53.34″
	SY0	120°34′08.70″	33°47′48.48″
	SY1	120°47′04.38″	33°47′48.48″
	SY2	121°00′00.00″	33°47′48.48″
	SY3	121°16′37.20″	33°47′48.48″
	SY4	121°35′45.60″	33°47′48.48″
	SF6	121°49′48.00″	33°47′48.48″
	SH5	121°30′00.00″	34°20′00.00″
	BH5	121°15′00.00″	34°20′00.00″
	BH4	121°00′00.00″	34°20′00.00″
	BH3	120°45′00.00″	34°20′00.00″
	BH2	120°30′00.00″	34°20′00.00″
	LYG5	120°31′16.02″	34°45′50.10″
	LYG4	120°13′05.94″	34°45′50.10″
	LYG3	119°53′56.22″	34°45′50.10″
	LYG2	119°42′05.76″	34°45′50.10″
	LYG1	119°30′17.16″	34°45′50.10″
	HZW2	119°22′48.00″	34°52′48.00″
	HZW4	119°31′32.88″	34°54′43.20″
	HZW1	119°18′00.00″	35°00′00.00″
	HZW3	119°21′00.00″	35°03′36.00″
	T4-2	119°54′10.26″	34°37′13.38″
	T4-1	120°06′14.76″	34°28′36.72″
	BH1	120°18′19.26″	34°20′00.00″
	T1-2	120°26′13.98″	34°03′54.24″
	T2-2	120°38′32.16″	34°03′54.24″
2024	LYG5	120°31′15.60″	34°45′46.80″
	HZW1	119°33′14.40″	34°57′21.60″
	HZW2	119°35′42.00″	34°52′58.80″
	HZW3	119°29′45.60″	34°57′21.60″
	HZW4	119°35′02.40″	34°54′36.00″
	T1-1	120°42′03.60″	33°33′54.00″
	T1-2	120°26′09.60″	34°03′54.00″
	T2-1	120°56′06.00″	33°33′54.00″
	T2-2	120°38′31.20″	34°03′54.00″
	T3-1	121°37′12.00″	33°13′40.80″
	T3-2	121°44′24.00″	33°26′24.00″
	T3-3	121°51′00.00″	33°38′24.00″
	T4-1	120°06′10.80″	34°28′33.60″
	T4-2	119°54′10.80″	34°37′15.60″
	RD1	121°20′31.20″	32°40′08.40″
	RD2	121°27′10.80″	32°39′57.60″
	RD3	121°45′00.00″	32°40′01.20″
	RD4	122°00′00.00″	32°39′57.60″
	RD5	122°15′00.00″	32°39′57.60″
	DF1	120°50′00.00″	33°19′40.80″
	DF2	121°05′20.40″	33°17′27.60″
	DF3	121°20′00.00″	33°19′40.80″
	DF4	121°34′01.20″	33°19′44.40″
	DF5	121°49′58.80″	33°19′44.40″
	SY0	120°34′08.40″	33°47′52.80″
	SY1	120°47′02.40″	33°47′52.80″
	SY2	121°00′00.00″	33°47′52.80″
	SY3	121°16′37.20″	33°47′49.20″
	SY4	121°35′45.60″	33°47′52.80″
	BH1	120°18′19.80″	34°20′02.40″
	BH2	120°30′03.60″	34°20′00.00″
	BH3	120°45′00.00″	34°20′02.40″
	BH4	121°00′00.00″	34°20′02.40″
	BH5	121°15′00.00″	34°20′02.40″
	LYG1	119°30′17.00″	34°45′47.00″
	LYG2	119°42′07.20″	34°45′50.40″
	LYG3	119°53′56.40″	34°45′50.40″
	LYG4	120°13′03.00″	34°45′50.40″

**Table 2:** Polymerase chain reaction (PCR) primers used in this study.

Primer	Primer Sequence	Reference
ITS-F	5′-TCTTTGAAACCGTATCGTGA-3′	[36]
ITS-R	5′-GCTTATTGATATGCTTAAGTTCAGCGGGT-3′	[37]
5S-F	5′-GGTTGGGCAGGATTAGTA-3′	[38]
5S-R	5′-AGGCTTAAGTTGCGAGTT-3′	

**Table 3:** Physical and Chemical Parameters of the Southern Yellow Sea in March 2023 and 2024.

Year	Station	Salinity		PH		DO (μmol/L)		COD-Mn (μmol/L)	
		Average Value	Range of Change	Average Value	Range of Change	Average Value	Range of Change	Average Value	Range of Change
2023	Surface layer	30.42 ± 1.12	27.69~32.13	8.14 ± 0.05	8.06~8.22	280.84 ± 8.47	257.19~295.00	16.14 ± 7.91	7.81~47.50
	Bottom layer	30.41 ± 1.09	27.69~32.09	8.15 ± 0.05	8.06~8.23	295.18 ± 6.91	285.63~315.63	23.36 ± 16.74	8.44~78.75
2024	Surface layer	29.54 ± 1.99	23.49~31.65	8.07 ± 0.06	7.97~8.20	292.48 ± 5.32	278.44~300.94	29.38 ± 11.60	17.19~69.06
	Bottom layer	29.89 ± 1.40	26.53~31.87	8.08 ± 0.06	7.98~8.20	296.46 ± 4.84	280.94~305.63	34.93 ± 15.76	20.94~86.56

## Data Availability

Data are available from the corresponding author upon reasonable request.
